# The Role of Single-Molecule Force Spectroscopy in Unraveling Typical and Autoimmune Heparin-induced Thrombocytopenia

**DOI:** 10.3390/ijms19041054

**Published:** 2018-04-02

**Authors:** Van-Chien Bui, Thi-Huong Nguyen

**Affiliations:** 1 Institute for Immunology and Transfusion Medicine, University Medicine of Greifswald, 17475 Greifswald, Germany; buivanchien@gmail.com; 2 ZIK HIKE—Center for Innovation Competence, Humoral Immune Reactions in Cardiovascular, 17489 Greifswald, Germany

**Keywords:** heparin- and antibody-induced thrombocytopenia, HIT, mechanism, binding force, PF4

## Abstract

For the last two decades, heparins have been widely used as anticoagulants. Besides numerous advantages, up to 5% patients with heparin administration suffer from a major adverse drug effect known as heparin-induced thrombocytopenia (HIT). This typical HIT can result in deep vein thrombosis, pulmonary embolism, occlusion of a limb artery, acute myocardial infarct, stroke, and a systemic reaction or skin necrosis. The basis of HIT may lead to clinical insights. Recent studies using single-molecule force spectroscopy (SMFS)-based atomic force microscopy revealed detailed binding mechanisms of the interactions between platelet factor 4 (PF4) and heparins of different lengths in typical HIT. Especially, SMFS results allowed identifying a new mechanism of the autoimmune HIT caused by a subset of human-derived antibodies in patients without heparin exposure. The findings proved that not only heparin but also a subset of antibodies induce thrombocytopenia. In this review, the role of SMFS in unraveling a major adverse drug effect and insights into molecular mechanisms inducing thrombocytopenia by both heparins and antibodies will be discussed.

## 1. Introduction

Thrombocytopenia affects 30–50% critically ill patients [1]. Heparin-induced thrombocytopenia (HIT), a severe adverse drug effect, occurs when patients receive polyanion anticoagulants like heparins to prevent and treat thromboembolic diseases. HIT is caused by the transient production of platelet-activating antibodies belonging to the IgG class that recognize multimolecular complexes formed by cationic platelet factor 4 protein (PF4, a positively charged small cytokine, released from alpha-granules of activated platelets) and polyanionic heparins.

Even though the activated platelets release a high level of PF4, the nonactivated platelets also produce PF4s at a certain low concentration. When patients receive heparins, some of them bind to PF4s to form ultra large PF4/Heparin (PF4/H) complexes [2]. This binding reaction induces a conformational change in PF4s [3,4,5] which results in an expression of new epitopes. The human immune system recognizes these neoepitopes on the complexes as “foreign”, and therefore, it produces antibodies against the PF4/H complexes (called aPF4/H Abs) [2]. The resulting complexes of PF4, heparin and a subset of aPF4/H Abs can become antigenic targets that bridge platelets [6,7] and blood cells [8]. Fragment antigen binding (Fab) part of an antibody can link to a PF4/H complex while its fragment crystallizable (Fc) region binds effectively to FcγRIIa receptor [9,10] on platelet membrane. This cross-linking leads to platelet aggregation and activation that release more PF4s and promotes the formation of additional ultra large immune complexes in blood. These complexes rapidly recruit other platelets into the prothrombotic process leading to the loss of platelets. Typically, 5–14 days after heparin exposure, platelet count is reduced to <15–20 × 10^9^ cells/L (platelet count decreases more than 50%) [11]. This can lead to a massive platelet activation and triggers a clotting cascade that results in thrombin generation and increases the risk for vessel occlusions such as venous thrombosis, myocardial infarction or stroke [4,12,13]. However, only long heparin can alter the structure of PF4 and induce antigenic PF4/H complexes [5,14,15]. Depending on the length of heparin, HIT occurs in ≤5% of patients receiving high molecular weight unfractionated heparin [16] or in ≤1% of patients receiving low molecular weight heparins [17,18].

Importantly, it has been recognized that some patients had clinical symptoms and laboratory features of HIT despite not having previously received heparin during the last decade [19]. This symptom is called autoimmune HIT (aHIT), which may follow an atypical clinical course [19,20]. Such patients often show unusual clinical features, such as severe thrombocytopenia (platelet count <20 × 10^9^ platelets/L). Patients with aHIT have aPF4/H Abs which can lead to symptomatic thrombocytopenia and excessive vascular thrombosis in the absence of heparin. The extreme sequela of these aPF4/H Abs is the development of multiple vessel occlusions without drug exposure. 

The typical HIT occurs only when patients receive heparin while aHIT occurs when patients do not receive heparin. Recently, characteristics of the interactions between PF4 and heparins in HIT [14,15,21] and detailed insights into binding mechanisms between molecules in aHIT have been unraveled [22,23]. In this review, the role of single-molecule force spectroscopy (SMFS) in unraveling a major adverse drug effect and mechanism inducing thrombocytopenia by both heparins and human-derived aPF4/H Abs at the single molecular level will be discussed. 

To date, only SMFS techniques allow direct measurements of bond dynamics and kinetic properties of interactions at single-molecular level [24,25,26,27]. SMFS techniques such as optical tweezers (optical traps), magnetic tweezers, atomic force microscopy (AFM), microneedle manipulation, biomembrane force probe, and flow-induced stretching have become important tools to directly unravel the insights into molecular interactions. SMFS provides novel information that could not be obtained by other bulk-related methodologies [28]. A high resolution in the range of angstrom in length and piconewton in force (5 pN–100 nN) can be gained by SMFS techniques [25,29,30,31]. Applying SMFS on the complexes, direct insights in the binding strength, transition states, energy landscape, and thermodynamic and kinetic parameters of many interactions such as ligands-receptors [32,33,34], proteins-cells [35,36,37], cells-cells [28,29,38,39,40] and even cell mechanics [41,42] could be unraveled [28,43,44]. 

SMFS-based atomic force microscopy in principle describes the force-displacement curves obtained by oscillating the scanner in z-direction, while the scanner movement in the x- and y-directions is disabled. In such a way, the deflection signal from the cantilever and the movement of the piezoelectric scanner are recorded. To measure interactions between a ligand and a receptor, the ligand (or receptor) is linked to an AFM-tip while the receptor (or ligand) is immobilized on the substrate. When the tip approaches the substrate, the interaction between ligand and receptor will occur and their binding force is measured when the tip separates from the substrate. To facilitate molecular interaction, a flexible linker (e.g., polyethylene glycol: PEG) is utilized to covalently link the molecules of interest to the tip/substrate [21,26,45]. The use of long spacers increase the molecular mobility, avoid non-specific tip/substrate interactions, distinguish specific and non-specific interactions, allow free molecular reorientation in 3-dimensional structures, reduce potential molecular denaturation, and protect molecules from the mechanical tip-substrate crush [46]. In such a way of setup, interactions of ligand and receptor could be measured. 

## 2. Insights into Binding Mechanisms of Typical HIT

### 2.1. Heparins and PF4 Associated with HIT

Heparins are the glycosaminoglycans containing glucosamine residues with a high degree of sulfation that dictates their biological activities [3,47,48]. It has been shown previously that the glycosaminoglycans support the sequestration of *Plasmodium falciparum*-infected red blood cells in the microvascular endothelium [49,50]. Their pharmacologic activity is mediated by a chemically unique pentasaccharide sequence present in about 30% of all heparin molecules. Heparin behaves like simple entropic spring forces, which is produced by sugar rings of heparin flipping to more energetic and more extended conformations [51,52]. Both low and high molecular weight heparins are available. The source of high molecular weight unfractionated heparin (UFH) influences the risk of HIT, i.e., bovine UFH is more likely to cause HIT than porcine UFH [53,54,55]. Besides UFH, low molecular weight heparins (LMWH) produced from UFH by chemical fractionation are widely used in clinical practice [56,57,58,59,60]. Due to their shorter chain length, LMWHs show weaker interaction with PF4 than UFH. The UFH and the PF4 form ultra-large complexes (ULCs) when both are present approximately at an optimal 1:1 ratio. Comparing with UFH, LMWHs form smaller complexes with PF4. ULCs showed a greater capacity to promote platelet activation than small complexes [61]. These differences in complex formation between UFH and LMWHs translate into their risk for inducing HIT in patients. Though LMWHs induce HIT about 10 times less frequent than UFH, HIT still randomly occurs during application of LMWHs [62,63,64,65]. 

### 2.2. Boundary between Antigenic and Nonantigenic Heparins

It has been shown that at least three bonds between the polyanion and PF4 in the PF4/polyanion complex should be formed in order to expose neoepitopes for binding of aPF4/H Abs [14]. When interacting with PF4, the quantity or density of sulfate groups on the polyanions determine their molecular effects [14]. In particular, the polyanions which bind to PF4 tetramer with less than three sulfate bonds are unable to expose neoepitopes [3,66]. The findings suggested an existence of a boundary between antigenic (risk for HIT) and non-antigenic heparins (non-risk for HIT). 

**Figure 1:**
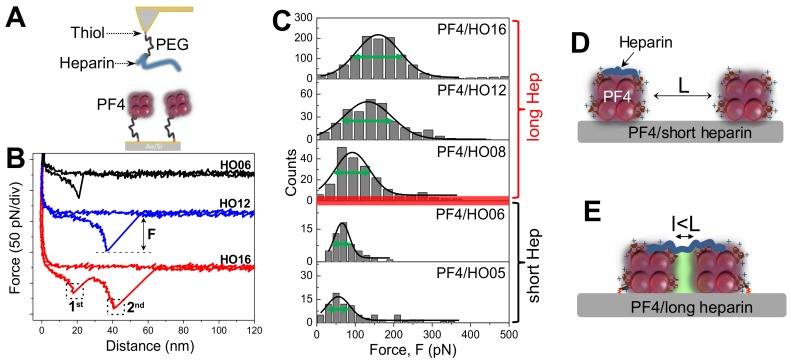
Insights into the interaction between platelet factor 4 (PF4) and heparins of different lengths. (**A**) Single-molecule force spectroscopy (SMFS) setup for measuring binding strength between heparins (blue) of different length immobilized on the tip and PF4 tetramers immobilized on the substrate *via* polyethylene glycol (PEG) linkers. (**B**) Short heparin (HO06, black) shows only one peak with low binding force (black), longer heparin (HO12, blue) also exhibits one peak with higher binding force (F), whereas longest heparin (HO16, red) displays two rupture peaks (1st and 2nd). (**C**) Gaussian fits of rupture force histograms (solid curves) show narrow widths (green arrows) of the force distributions for short heparins ≤6-mer and wider widths for long heparins ≥8-mer, suggesting different binding characteristics at 8-mer (red bar). (**D*,*E**) Models describing different binding pathways of short (**D**) and long (**E**) heparins with two PF4 tetramers away from each other in a distance L. When the AFM tip approaches the substrate, the short heparin binds to one PF4, while long heparin bridges two PF4s and forces them closer to each other in the distance l < L, merging two hydrophobic surfaces (green shaded area).

Recent studies by SMFS have identified this boundary [21]. By immobilizing LMWH heparin of different lengths on AFM-tips and PF4s on the substrates (Figure 1A) and analyzing the interaction forces (Figure 1B), it is found that both numbers of specific rupture events and magnitude of rupture forces rise with increasing heparin length, suggesting long heparins formed more bonds with PF4 than short ones [21]. 

In addition, the variation of the rupture forces obtained by long heparins ≥8-mer is larger than that obtained by short ones ≤6-mer (Figure 1B). When applying the Bell–Evans model [67] to the rupture forces measured at different loading rates, short heparins (≤6-mer) show higher k_off_ values than long heparins (≥8-mer), suggesting that complexes between PF4 with long heparins are more stable than those with short heparins. Consistently, the thermal on-rate of the interaction shows that short heparins bind to PF4s with ~10–20-times faster than long heparins [3]. Thus, SMFS results show a clear different feature between short and long heparin at ~8-mer. 

The suggested heparin boundary by SMFS results has been further proven by multiple techniques including isothermal titration calorimetry (ITC) [3], circular dichroism (CD) spectroscopy and enzyme-linked immunosorbent assay (ELISA) [4]. By ITC, Kreiman and coworkers obtained a rise in enthalpy of the reaction with an increase of heparin length and reach a maximum at ~11-mer [68]. Consistently, CD spectroscopy, a sensitive methodology to the secondary structure and folding properties of proteins [69], shows a change in β-sheet content in PF4/H complexes ≤30% for short heparin and >30% for long heparins. In the meanwhile, the ELISA for detecting binding of aPF4/H Abs via optical density (OD), shows OD ≤ 0.5 for short heparin and >0.5 for longer heparins (>8-mer) [3,21]. The OD of 0.5 is the threshold to determine whether a heparin used in the ELISA was able to support binding of aPF4/H Abs. 

The boundary between antigenic and non-antigenic heparin was finally determined between 8- and 11-mer [3,21]. These findings are particularly important to understand PF4-Heparin binding processes and to develop new heparin-derived drugs with reduced risk for adverse immune reactions. 

### 2.3. Long Heparin-Induced Additional Bond among PF4 Molecules

The magnitude of adhesion force in SMFS measurement provides information on the binding strength of PF4 to heparins of different lengths. Analysis of features of the force-distance curves also provides additional important information of the interactions. Nguyen and coworkers [21] observed that short heparins exhibit only ‘one rupture step’ when interacting with PF4, whereas long heparins show two rupture steps (Figure 1B). The difference in ‘rupture step’ is attributed to the dissimilar binding pathways between short and long heparins. By analyzing binding forces of the interactions between PF4 and heparins and between PF4 and PF4, it has been found that short heparins form only one bond with PF4, whereas long heparins form one bond with PF4s and trigger additional PF4-PF4 bond [21]. Even though the concept of the PF4-PF4 bond, in general, cannot be accepted because PF4s are highly positive proteins, and therefore, strongly repel each other. However, PF4-Heparin interactions are more complex than general ligand-receptor interactions which are attributed to the electrostatic attraction. When forming a complex with a highly negative charged heparin, the positive charged PF4 is probably neutralized that results in an emergence of two hydrophobic PF4 surfaces [66]. Based on these findings, a model for PF4-heparin interaction has been proposed (Figure 1D,E). Due to their sizes, the short heparins simply bind to a single PF4 tetramer (Figure 1D), whereas the long heparins neutralize positive charges on PF4 tetramers and switch the charges between two PF4 tetramers from repulsion to attraction. Heparin reacts as a catalyst that forces two PF4 molecules close to each other within a distance l (l < L), resulting in two merged hydrophobic PF4 surfaces (Figure 1E). This way of interaction results in extremely stable PF4/H complexes, especially for long heparins. 

## 3. Insights into Binding Mechanism of aPF4/H Abs-Induced Autoimmune HIT

### 3.1. Characteristics of aPF4/H Abs

**Figure 2:**
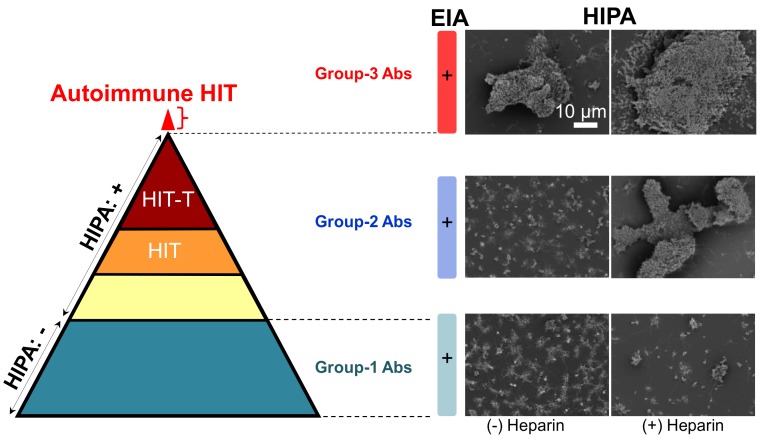
Reaction patterns of aPF4/H Abs. (**Left**) The pyramid shows antibodies of three groups. They are all positive in ELISA. Group-1 Abs (dark cyan) do not activate platelets in a functional assay (−). group-2 Abs contain different types of antibodies, i.e., some do not induce HIT (yellow), some induce HIT (orange), and some induce HIT with thrombosis (brown). Recent studies found an additional small subset of patient’s content autoimmune group-3 Abs (red). (**Right**) Scanning electron microscopy images showing platelet aggregates in the presence (+) or absence (−) of heparin. Group-1 Abs induce only small aggregates (bottom panel) reflecting the background platelet activation, group-2 Abs (middle panel) cause large aggregates only in the presence of heparin, and group-3 Abs induce large aggregates even in the absence of heparin. Scale bar is applied to all images. Adapted from [7,22].

All human-derived aPF4/Polyanion (PF4/P) antibodies bind to immobilized PF4/P complexes in ELISA [15], but only some of them activate platelets in functional assays, i.e., the heparin-induced platelet activation assay (HIPA) [15] or the serotonin release assay (SRA) [70,71]. Human-derived aPF4/H Abs positive in ELISA are composed of three groups, i.e., group-1 is negative in HIPA; group-2 is positive in HIPA but requires heparin; group-3 is positive in HIPA even in the absence of heparin as summarized in Figure 2. Group-3 Abs developed from patients who had clinical autoimmune thrombocytopenia, and therefore, they are defined as ‘autoantibodies’ [72].

Insights into the reactions of aPF4/H Abs with PF4/H complexes may lead to clinical visions especially may help to better understand general mechanisms of antibody-mediated autoimmunity HIT. In contrast to the detailed characterization of the PF4/H complexes, little is known about features of aPF4/H Abs in the pathogenesis of HIT due to a difficulty in subtracting the pathogenic HIT antibodies directly from human sera. This is because both pathogenic and non-pathogenic antibodies bind to the PF4/H antigen. Nevertheless, Newman and coworkers reported that aPF4/P Abs can be purified by PF4-agarose beads [8]. Later, Amiral and coworkers described that affinity purification of aPF4/P Abs resulted in a mixture of IgA, IgM, and IgG [73]. In this mixture, only a subset of IgG antibodies activates platelets [70]. The mixture of different type of antibodies increases the difficulty in characterizing aPF4/P Abs at a single molecular level using SMFS. To overcome this limitation, two-step affinity chromatography has currently established to isolate aPF4/H Abs from HIT patients’ sera. By this method, individual groups of aPF4/P Abs from patients’ sera were successfully isolated. The purified Abs showed similar characteristics to the original serum in both ELISA and HIPA. Titrating the antibodies in ELISA show an increase in OD value when antibody concentration increases. OD was highest for group-3, lower for group-2 and lowest for group-1 Abs. In the HIPA test, group-1 Abs did not cause platelet aggregation up to 89.7 µg/mL; group-2 Abs induced platelet aggregation at ≥43.5 µg/mL, but only in the presence of heparin; while group-3 Abs induced platelet aggregation at ≥5.2 µg/mL even in the absence of heparin. These results are consistent with previous findings [74,75].

### 3.2. Bond Dynamics of aPF4/H Abs

**Figure 3:**
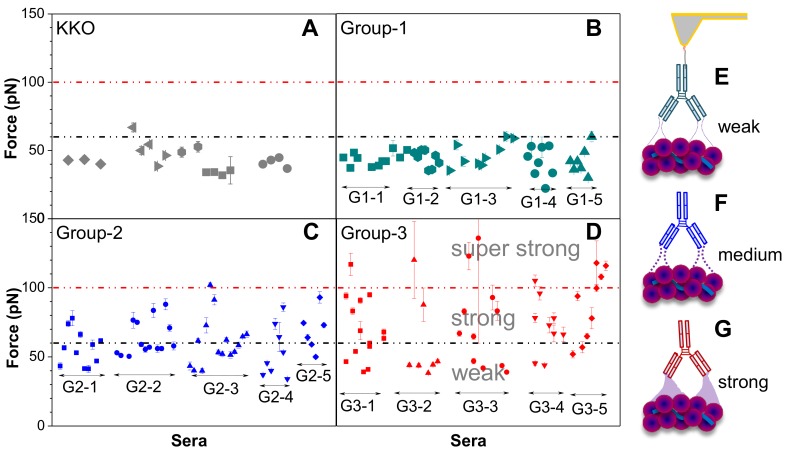
Binding strength of aPF4/H Abs to PF4/H complexes. Each dot shows the mean and standard error of the rupture force for each respective antibody from five sera per group. (**A**) KKO and (**B**) group-1 Abs bind to PF4/H complexes with a binding strength mostly ≤60 pN (black dotted line), while (**C**) group-2 and (**D**) group-3 Abs consist of Abs with different binding forces. Cartoons show weak (**E**), medium (**F**) and strong (**G**) interaction force of KKO/group-1, group-2 and group-3, respectively, when interacting with PF4/H complexes. Adapted from [22].

The binding strengths between antibodies and PF4/H complexes were determined by retracting the antibodies coated AFM-tips away from PF4/H complexes coated substrates. The mouse monoclonal antibody named KKO mimics the biological activity of human of aPF4/H Abs [76], causes HIT in an animal model in vivo [77,78], and has been used as a standard to understand the binding characteristics of aPF4/H Abs [31,76]. Weakest binding forces (Table 1) were obtained for KKO (43.6 ± 8.8 pN, Figure 3A) and group-1 Abs (44.0 ± 8.1 pN, Figure 3B), higher for group-2 Abs (60.6 ± 15.4 pN, Figure 3C) and highest for group-3 Abs (72.4 ± 26.2 pN, Figure 3D), indicating that groups 2–3 Abs bind to the complexes stronger than KKO and group-1 Abs [22]. However, group-2 Abs contain different types of antibodies as observed by a large variation of all binding forces (~40% exceeded 60 pN) (Figure 3C). For group-3 Abs, the variation of binding force is even higher than that of group-2 Abs as shown by ~44% of all binding forces ≥60 pN and ~15% even exceeded 100 pN (Figure 3D). The low variability in binding forces of KKO and group-1 Abs has been attributed to the fact that they contain homogeneous antibodies, whereas patient’s sera such as group-2 and group-3 Abs contained polyclonal mixtures of aPF4/P Abs ranging from weak, strong to super strong reactions. Among these Abs, group-2 Abs contain reacting antibodies like group-1 Abs, while group-3 is highly complicated as it is composed of not only antibodies reacting like group-1 (weak binding, Figure 3E) and group-2 Abs (medium binding, Figure 3F) but also some additional super strong reactive (strong binding, Figure 3G) antibodies. The aPF4/H Abs also show different reactivity patterns under various pH and ionic strength conditions [79]. 

Consistently, reaction of group-3 Abs bound to PF4/H complexes with much higher heat (ΔH = −2.87 ± 2.06 × 10^8^ cal/mol) than group-2 Abs (ΔH = −2.90 ± 0.4 × 10^4^ cal/mol), and their dissociation constant (K_D_) (~5.3 nM) was about two orders of magnitude lower than that of group-2 Abs (~1.7 × 10^2^ nM) [22]. Besides that, the group-3 Abs have the highest binding affinity (k_off_ = 0.12 s^−1^) as compared with group-1 Abs (k_off_ = 15.6 s^−1^), group-2 Abs (k_off_ = 2.0 s^−1^), or KKO (k_off_ = 2.2 s^−1^) (Table 1). The lowest thermal off-rate specifies that multiplexes induced by PF4/H complexes with group-3 Abs are more stable than those formed with other antibody groups.

### 3.3. Autoimmune HIT (Group-3) Antibodies Cluster PF4 and Allow Binding of Other aPF4/H Abs

**Figure 4:**
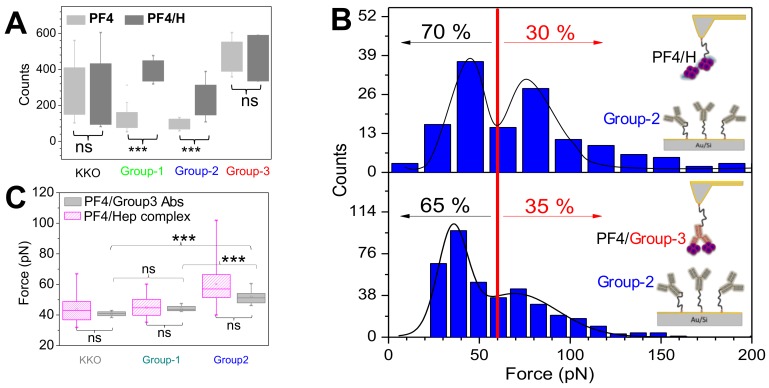
PF4/group-3 Abs complexes expose binding epitopes for other aPF4/H Abs. (**A**) Binding events when group-1 and group-2 Abs interacted with PF4 (light gray) was far different from those with PF4/H complexes (dark gray) while the standard KKO and group-3 Abs did not show a significant difference (ns). (**B**) The group-2 Abs immobilized on the substrates interacted with PF4/H complexes (top) showing a similar feature of force distribution as compared with that of the PF4/group-3 Abs complexes (bottom): ~70% of binding forces were ≤60 pN and ~30% of binding forces ≥60 pN. (**C**) The summary counts of interactions with binding forces >60 pN between either PF4/group-3 antibody complex (gray) or PF4/H complexes (pink) with KKO, group-1, or group-2 Abs again underscores that group-3 Abs can form complexes with PF4 which allow binding of other aPF4/H Abs. Statistic obtained by ANOVA tests show significant (***) or no significant (ns) difference. Adapted from [22].

The binding energy generated by the interaction of group-3 Abs with PF4 [22] was about four orders of magnitude higher than the energy released when a 16-mer heparin interacts with PF4 obtained by ITC [15]. As 16-mer heparin can force two PF4 molecules together, high energy release in the reaction of the group-3 Abs indicates that these Abs can also force two PF4 tetramers together and change PF4 conformation as shown by the negative entropy [22] These results indicate that there are the existence of PF4/group-3 antibody complexes in some patients while. For the known system, it has been clearly proved that the PF4/H complexes allow binding of pathogenic aPF4/H Abs. For the new finding system, it rises an intriguing hypothesis that the PF4/group-3 antibody complexes also allow binding of other aPF4/H Abs and activate platelets in the absence of heparins. This hypothesis was clearly proved by applying various methodologies [22]:

The first and center methodology was SMFS. By immobilizing aPF4/H Abs on AFM-tips and allowing them to interact with PF4 alone or PF4/H complexes coated on the substrates, group-1 and group-2 Abs showed much less binding events to PF4 than to PF4/H complexes, while a subset of group-3 Abs showed similar binding interactions (Figure 4A). The results indicate that only group-3 Abs can cluster PF4. The conclusion from SMFS data was clearly proved by dynamic light scattering (DLS), ITC and affinity purification of aPF4/H Abs. DLS results show that the size of PF4 suddenly increases when group-3 Abs are added. Consistently, ITC experiments showed strong interaction between group-3 and PF4, while other Abs do not show any interaction. While other antibodies required PF4/H complexes coated beads to be isolated from human sera, group-3 Abs can be purified directly from PF4 coated bead. Hardly any PF4/P Abs were obtained from control and group-1 sera whereas group-2 sera showed a minimal increase in IgG yield. The results indicate that a single antibody (group-3) can cluster PF4 molecules.

The next question is that if the PF4/group-3 antibody complexes also allow binding of other aPF4/H Abs to form antigenic complexes and activate platelets. To understand this, the cantilevers coated with group-3 Abs that showed high rupture forces with PF4/H complexes (≥80 pN), were selected and incubated with PF4 in the fluid phase to form PF4/group-3 antibody complexes (Figure 4B). The KKO, group-1 or group-2 Abs were immobilized on the substrates for measuring the interactions with PF4/group-3 Abs or PF4/H complexes immobilized on the cantilevers. The results showed no significant difference in binding strength between PF4/group-3 complexes and PF4/H complexes when interacting with the same type of antibody (Figure 4C). No significant difference in the binding force between PF4/group-3 Abs and PF4/H complexes when interacting with KKO, group-1 and group-2 Abs (Figure 4B) determined by SMFS rises a hypothesis that PF4/group-3 Abs complexes also allow binding of other aPF4/H Abs in the similar way PF4/H complexes do. 

The hypothesis from the SMFS results was then further proved by ELISA and DLS. The PF4 or PF4/Heparin ELISA shows that group-3 Abs bound quite strong to PF4 while other antibodies did not even though all Abs bound much stronger to PF4/H complexes than to PF4 alone. DLS showed that the group-3 Abs formed the largest complexes with PF4 as compared to other antibody groups, even larger than PF4/H complexes. The results from different techniques proved the hypothesis developed from SMFS results that group-3 Abs cluster PF4 and then allow binding of group-2 Abs in the same way as polyanions do [22].

**Figure 5:**
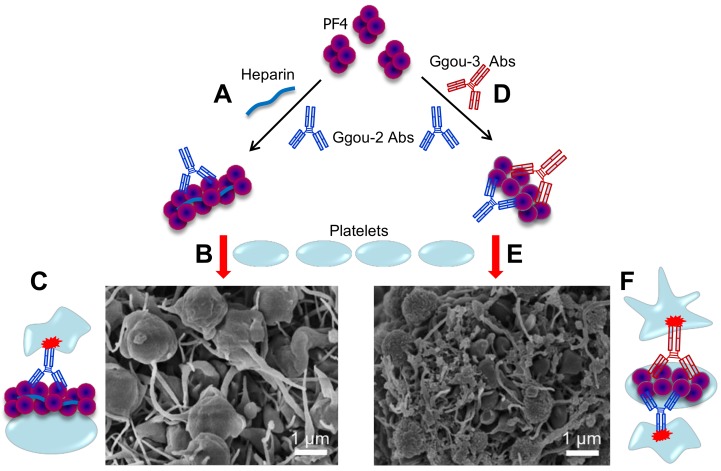
Typical HIT vs. autoimmune HIT. (**A**) PF4 form large complexes with heparin and the resulting PF4/H complexes allow binding of group-2 Abs blue which will then bridge platelets to form large platelet aggregates and activate them (**B**) via Fc receptor ((**C**), red), resulting of a typical HIT. (**D**) A subset of group-3 Abs (red) mimic heparin in clustering PF4, forming PF4/Group-3 antibody complexes which also allow binding of group-2 Abs, inducing platelet aggregation and activation (**E**), resulting of autoimmune HIT (**F**) Complex formed by group-3 antibody contains at least two pathogenic antibodies, both bind to platelet membrane, and therefore, enhanced platelet activation. Autoimmune HIT by group-3 Abs induces stronger platelet aggregation/activation as evidenced by tighter and denser aggregates than typical HIT formed by heparin and group-2 Abs (**B**). Adapted from [22,80].

Altogether, PF4 forms large complexes with heparin and allows group-2 Abs to bind and induce platelet aggregation/activation (Figure 5A–C). Importantly, a subset of group-3 Abs cluster PF4 and the resulting PF4/Group-3 antibody complexes also allow binding of group-2 Abs and enhance platelet aggregation/activation even stronger than heparins do, as shown by tighter and denser aggregates (Figure 5D–F).

## 4. Conclusions

Far from previous reports that SMFS is a powerful methodology to study the interaction/bond dynamics between two single molecules, cells or bacteria at single molecular level, this review emphasized that SMFS plays an important role directly in identifying a new mechanism of autoimmune HIT caused by a subset of anti-PF4/Heparin antibodies, which was not so far easily done by other bulk-related methodologies. SMFS provided an initial hypothesis that ‘not only heparin but also a subset of anti-PF4/Heparin antibodies induced thrombocytopenia in patients without heparin exposure’. This initial hypothesis could be simply proved by other methodologies. Even though SMFS is not a technique for clinical routine tests, it provided a higher accuracy in detecting pathogenic aPF4/H Abs than the recent clinical diagnostic PF4-ELISA, which shows only ~50% accuracy. Even though, the SMFS method exhibits several limitations such as being a time-consuming, low throughput and complicated method, application of SMFS in disease detection becomes more and more promising. To avoid speculation developed by SMFS analysis, using other complementary methods to support the hypothesis are highly effective. The recently reported on autoimmune thrombocytopenia induced by a subset of human-derived antibodies may have major implications for understanding other autoimmune disorders in hemostasis or detection of other diseases.

## Figures and Tables

**Table 1:** Binding characteristics of aPF4/H antibodies.

Antibody	F(pN)	k_off_ (s^−1^)	Cluster PF4
KKO	43.6 ± 8.8	2.2	weak
Group-1	44.0 ± 8.1	15.6	no
Group-2	60.6 ± 15.4	2.0	weak
Group-3	72.4 ± 26.2	0.12	strong

## Abbreviations

HIT	Heparin-induced thrombocytopenia
PF4	Platelet factor 4
H	Heparin
PF4/H	Platelet factor 4/Heparin
aPF4/H Abs	anti-PF4/Heparin antibodies
PF4/P	Platelet factor 4/Polyanion
HO05	Fondaparinux
HO06	6-mer heparin
HO08	8-mer heparin
HO12	12-mer heparin
HO16	16-mer heparin
UFH	Unfractionated heparin
KKO	Mouse monoclonal antibody that recognizes human platelet factor 4
AFM	Atomic force microscopy
SMFS	Single-molecule force spectroscopy
ELISA	Enzyme-linked immunosorbent assay
PEG	Polyethylene glycol
HIPA	Platelet activation assay
DLS	Dynamic light scattering
ITC	Isothermal titration calorimetry
CD	Circular dichroism
OD	Optical density
